# Is increasing patient participation in rheumatoid arthritis disease management the key to better treatment adherence?

**DOI:** 10.1093/rap/rkac022

**Published:** 2022-03-29

**Authors:** Gouri M Koduri

**Affiliations:** Rheumatology Department, Southend University Hospital, Westcliff on Sea, Essex, UK

Early intensive treatments for RA, including combination therapy, are being used to achieve remission or a low disease activity state. Furthermore, it is increasingly recognized that decision-making should be balanced and shared between the patient and the clinician.

The consequences of non-compliance are significant. Uncontrolled active RA causes joint damage, disability, decreased quality of life and co-morbidities [1], hence it is imperative to improve the consultation process, which can potentially impact treatment adherence. Studies have shown that DAS (DAS28) and outcomes are significantly lower in patients with good adherence to treatment [2].

## Benefits of patient participation

A well-informed patient is more likely to accept and adhere to treatment. This not only improves outcomes but also heightens patient satisfaction. The treat-to-target and the EULAR recommendations include patient-centred care, non-pharmacological support and psychosocial support. They also emphasize education and empowering the patient to engage in shared decision-making with their clinician [3].

A survey exploring the treatment experience of RA patients, carried out by the National Rheumatoid Arthritis Society (NRAS) [4], in association with medac Pharma, found that >70% of RA patients stopped taking their oral MTX, increasing the risk of flares and relapses. Furthermore, poor adherence to MTX is linked to the patients’ lack of understanding and involvement in their treatment choice.

These new insights, within the context of the first National Institute of Health and Care Excellence (NICE) Shared Decision-making guidance [5], have instigated the need to examine whether greater patient participation in RA disease management could ensure patient preference and improve adherence rates and outcomes.

The NRAS survey results found that only 9% of RA patients prescribed the first-line treatment were given a choice regarding the route of administration, and nearly all of them (95%) were first prescribed oral MTX. One-fifth of patients reported that they received little to no information regarding its benefits or possible side effects. The primary impact upon treatment adherence was found to be unpleasant gastroenterological side effects contributing to treatment discontinuation in >45% of patients [4].

## Improving treatment choice for RA patients for better adherence and outcomes

Sustained remission or low disease activity with minimal side effects is the goal of RA treatment, yet there remains some debate concerning the optimal route for MTX, step-up strategy and combination therapy. MTX has been the gold-standard treatment to control inflammatory arthritis since the 1980s in most countries. Owing to its various routes of administration and versatility of doses available, it can be used as a monotherapy and in combinations [6]. Yet, despite its efficacy and safety, average adherence rates to MTX are relatively poor, between 30 and 66% [7, 8]. The British Society for Rheumatology (BSR) recommends that patients on MTX are co-prescribed a minimum dose of 5 mg folic acid once a week. However, there has been much discussion regarding the dose of folic acid and whether it has benefits in reducing toxicity without decreasing MTX efficacy.

It is well recognized that the clinical efficacy of s.c. MTX is superior to oral MTX at the same dose, but real-world evidence suggests that it is under-utilized. Injectable MTX might have been used less frequently because of functional limitations and concerns about needle-stick safety. However, the introduction of prefilled s.c. MTX auto-injectors overcomes many of these concerns. The recent NRAS patient experience survey sheds light on patient preferences, with as many as 75% subsequently switching from oral to injectable MTX. Forty-two per cent experienced a significant reduction in their side effects, and nearly 50% reported a positive impact on their QOL [4]. There are no studies specifically comparing oral *vs* s.c. MTX; therefore, direct medical costs and outcomes remain unknown. However, s.c. MTX in appropriate patients can shift from higher- to lower-cost treatment pathways, and further research is clearly needed. The cost benefits are also considerable. A study from the UK has shown that routine use of s.c. MTX after oral MTX failure has the potential to save an estimated £7000 per patient in the first year of therapy and £9 million per year nationally in new patients [9], compared with biologics.

Typically, the treat-to-target strategy can involve a trial of multiple DMARDs and biological agents. Triple therapy is less commonly used in clinical practice as a first-line treatment after MTX failure, although triple therapy has been promoted for many years. A study in Sweden showed that the use of infliximab cost €20 916 more than triple therapy and gained only a 0.01 increase in quality adjusted life years (QALYs) [10]. Few studies demonstrated that triple therapy was non-inferior to biologic therapy and there was a higher risk of infections with biologics. The risk and benefits of various treatment choices should always be discussed with patients. The economic implications might be obvious, but patient preference and safety remain paramount.

## Patient education and involvement

Education and empowerment augment treatment adherence. A patient who is well educated about their disease, potential side effects and the importance of adherence is well equipped to self-manage their condition and make better treatment choices. The duration of consultation time also impacts treatment response. According to the NRAS survey, patients who had longer consultations reported a more positive response to their treatment [4]. Adopting the ASK (address, share, know) approach, together with shared decision-making tools, can help to structure the consultation and help patients to obtain the information they need within the time allocated. It enables patients to address their most pressing concerns during the consultation, including side effects and other available options, which leads to better treatment outcomes. At Mid and South Essex Hospitals, as in many RA clinics, after a diagnosis, patients are seen within a week for education and to give them their medication. An education session is facilitated with a small group of patients, providing an ideal opportunity to discuss concerns and to learn about RA, flares, MTX, diet and exercise.

From the perspective of a clinician, it is important to optimize RA treatment to maximize clinical outcomes and minimize economic costs. However, recent research has shown the importance of patient understanding and involvement in the decision-making process to achieve improvements on overall health, adherence and outcomes.

*Funding:* No specific funding was received from any bodies in the public, commercial or not-for-profit sectors to carry out the work described in this article.

*Disclosure statement:* The author has declared no conflicts of interest.

## Data availability statement

The data underlying this article will be shared on reasonable request to the corresponding author.

## References

[1] Scott DL , Wolfe F, Huizinga TWJ. Rheumatoid arthritis. Lancet 2010;376:1094–108. 20870100 10.1016/S0140-6736(10)60826-4

[2] National Rheumatoid Arthritis Society. Keep taking the pills. Available at: http://nras.org.uk/resource/keep-taking-the-pills/

[3] Hill J , Bird H, Johnson S. Effect of patient education on adherence to drug treatment for rheumatoid arthritis: a randomised controlled trial. Ann Rheum Dis 2001;60:869–75. 11502614 PMC1753835

[4] The RA and Adult JIA patient experience survey was carried out in March 2021 on behalf of medac Pharma in association with the National Rheumatoid Arthritis Society (NRAS). 589 UK-based patients took part who had been diagnosed within the last 5 years. Available at: https://nras.org.uk/resource/nras-patient-experience-survey-results-may-2021/

[5] NICE shared decision-making guidance – Published: 17 June 2021. Available at: https://www.nice.org.uk/guidance/ng197

[6] Senbel E , Tropé S, Herman-Demars H et al Benefits of switch from oral to subcutaneous route on adherence to methotrexate in patients with rheumatoid arthritis in real life setting. Patient Prefer Adherence 2021;15:751–60. 33888978 10.2147/PPA.S301010 PMC8055372

[7] Scheiman-Elazary A , Duan L, Shourt C et al The rate of adherence to antiarthritis medications and associated factors among patients with rheumatoid arthritis: a systematic literature review and meta-analysis. J Rheumatol 2016;43: 512–23. 26879354 10.3899/jrheum.141371

[8] Shea B , Swinden M, Tanjong Ghogomu E et al Folic acid and folinic acid for reducing side-effects in patients receiving methotrexate for rheumatoid arthritis (review). Cochrane Database of Syst Revs 2013;5:CD000951. 10.1002/14651858.CD000951.pub2 PMC7046011 23728635

[9] Fitzpatrick R , Scott DGI, Keary I. Cost-minimisation analysis of subcutaneous methotrexate versus biologic therapy for the treatment of patients with rheumatoid arthritis who have had an insufficient response or intolerance to oral methotrexate. Clin Rheumatol 2013;32:1605–12. 23835658 10.1007/s10067-013-2318-z

[10] van Vollenhoven RF , Ernestam S, Geborek P et al Addition of infliximab compared with addition of sulfasalazine and hydroxychloroquine to methotrexate in patients with early rheumatoid arthritis (Swefot trial): 1-year results of a randomised trial. Lancet 2009;374:459–66. 19665644 10.1016/S0140-6736(09)60944-2

